# Detailed analysis of low temperature inactivation of respiratory syncytial virus

**DOI:** 10.1038/s41598-024-62658-z

**Published:** 2024-05-23

**Authors:** Yuki Kitai, Oshi Watanabe, Suguru Ohmiya, Tomoko Kisu, Reiko Ota, Kazuyoshi Kawakami, Hiroshi Katoh, Kaori Fukuzawa, Makoto Takeda, Hidekazu Nishimura

**Affiliations:** 1 Department of Microbiology, Graduate School of Medicine and Faculty of Medicine, The University of Tokyo, Tokyo, Japan; 2 Virus Research Center, Clinical Research Division, Sendai Medical Center, Sendai, Miyagi Japan; 3 Department of Medical Microbiology, Mycology and Immunology, Tohoku University Graduate School of Medicine, Sendai, Miyagi Japan; 4 Graduate School of Pharmaceutical Sciences, Osaka University, Suita, Osaka Japan

**Keywords:** Virus structures, Virology, Viral membrane fusion

## Abstract

Our previous findings indicated that many respiratory syncytial virus (RSV) isolates are unstable at 4 °C compared to 20 °C. Some of the strains completely lose infectivity after 24 h at 4 °C. This study analyzed the inactivation process at 4 °C using a representative strain, RSV/Sendai/851/13. After 24 h of storage at 4 °C, the virus was completely inactivated but retained its ability to attach to and to be taken into host cells. It suggested a reduced fusion ability between the viral and cellular membranes. During storage at 4 °C, the RSV fusion (F) protein underwent a conformational change and was no longer recognized by pre-fusion form-specific antibodies. When the RSV/Sendai/851/13 strain was passaged at 4 °C, a variant with an amino acid substitution, I148T, in the F protein fusion peptide was selected. Also, an amino acid change in G protein demonstrating stability at low temperatures was obtained. These results show that the inactivation of RSV at 4 °C is due to the loss of membrane fusion activity in the F protein, which cannot maintain its pre-fusion state at 4 °C.

## Introduction

Respiratory syncytial virus (RSV), a negative-strand RNA virus from the Pneumoviridae family, is a significant pathogen of the upper respiratory tract and often causes severe bronchiolitis and pneumonia in infants, toddlers, immunocompromised patients, and older people^1–3^. In 2023, the Food and Drug Administration (FDA) approved RSV vaccines, while efficient therapeutic agents are not available^4,5^.

RSV comprises fusion (F) protein, attachment glycoprotein (G), small hydrophobic (SH) protein on the virion envelope, and nucleocapsid (N) protein, matrix (M) protein, large (L) protein, M2-1 protein, M2-2 protein, and phosphoprotein (P) inside the virion^6,7^. RSV attaches to the host cell surface via G protein and fuses with the cellular membrane at the cell surface or the endosomal membrane inside the cell via F protein, releasing viral RNA into the cytoplasm^6,7^. Membrane fusion is driven by the conformational change of the F protein from its pre-fusion to post-fusion form. Viral proteins and RNA are synthesized intracellularly and assembled at the cell surface to form progeny virions by budding^8^.

The Virus Research Center, Sendai Medical Center, Japan, has been isolating respiratory viruses, including RSV, from clinical specimens for decades, contributing to the diagnosing and surveillance of infectious diseases in the community^9–13^. A significant advantage of isolating infectious viruses from patients is that they can be used to analyze pathogenicity and antigenicity, and to evaluate the efficiency of antiviral drugs. However, viruses may lose infectivity during storage. RSV, especially, is temperature-labile: it may lose its infectivity when stored at 20 °C by heat; freezing-and-thawing also decreases its infectivity. Therefore, storage at 4 °C is usually recommended to prevent inactivation for short-term storage^14^.

Contrary to that perception, our previous study showed that many RSV isolates are actually less stable at 4 °C compared to 20 °C; in extreme cases, strains completely lose their infectivity when stored at 4 °C for 24 h^13^. Although the infectivity of RSV laboratory strains declines slightly during incubation at 4 °C^15^, no study has reported extreme virus inactivation at this temperature. This study aims to analyze the molecular basis of RSV inactivation at 4 °C.

## Results

### Effect of 4 °C treatment on infectivity, adsorption and uptake

**Figure 1:**
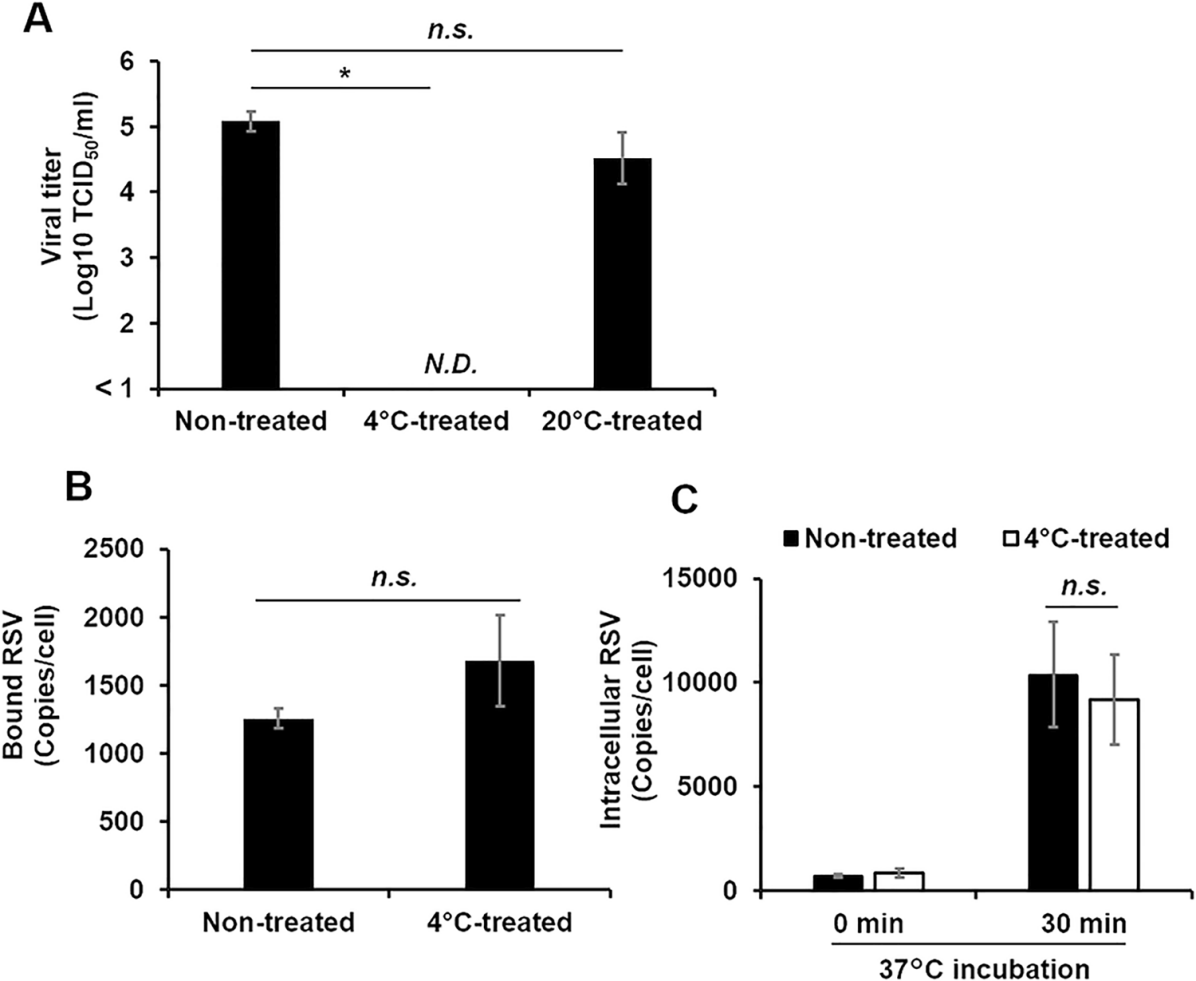
Effect of storage at 4 °C on virus infectivity, adsorption, and uptake into cell. RSV was incubated at 4 °C or 20 °C for 24 h and inoculated onto HEp-2 cells. Viral titer, adsorption, and uptake into cells were measured. For the control, RSV was inoculated onto HEp-2 cells without treatment at any temperature. (a) The viral titer was measured by the TCID_50_ method. Each column represents the mean ± SEM of three independent experiments. The amounts of virus adsorbed on and uptaken inside the cells were measured by quantitative real-time PCR. (b) The amount of virus adsorbed onto the cell surface. (c) The amount of virus inside the cells. The closed bar and open bar indicate non-treated and 4 °C-treated virus, respectively. The mean ± SD of three wells has been shown. *, *p* < 0.05; n.s., not significant; N.D., not detected; SEM, standard error of the mean; SD, standard deviation. The significant differences were determined by *t*-tests.

A representative strain, RSV/Sendai/851/13, was used in this study. RSV/Sendai/851/13 infectivity was examined after storage at 4 °C for 24 h. The infectivity decreased from 1.0 × 10^5^ median tissue culture infectious dose (TCID_50_)/ml to less than the detection limit (no cytopathic effect; CPE). In contrast, it was unaffected after storage at 20 °C (Fig. 1a), as reported in our previous study^13^. The first step of RSV infection is adsorption to the cellular surface^6^. Virions adsorbed to the HEp-2 cell surface were quantified by a previously published method using quantitative PCR^16^. The amount of RSV virions adsorbed to the cell surface did not differ significantly between the 4 °C-inactivated and non-treated virus (Fig. 1b). Virion uptake into the cells following adsorption was also evaluated using a previously published method using quantitative PCR^17^. Briefly, after adsorption of RSV to the cells at 4 °C, the virus was allowed to enter the cells at 37 °C. The viral RNA in the cells was quantified after removing the virus particles remaining on the cell surface by trypsin treatment. The efficient removal of virions from the cell surface by trypsin treatment was verified (Fig. S1). The level of virions taken up by the cells (assessed by the amount of viral RNA in the cells after incubation at 37 °C for 30 min) did not differ significantly between the 4 °C-inactivated treated and non-treated virus (Fig. 1c). These results indicate that the inactivation of RSV at 4 °C is not due to the failure of viral attachment or virus uptake.

### Loss of intracellular viral protein synthesis of an RSV strain at 4 °C

**Figure 2:**
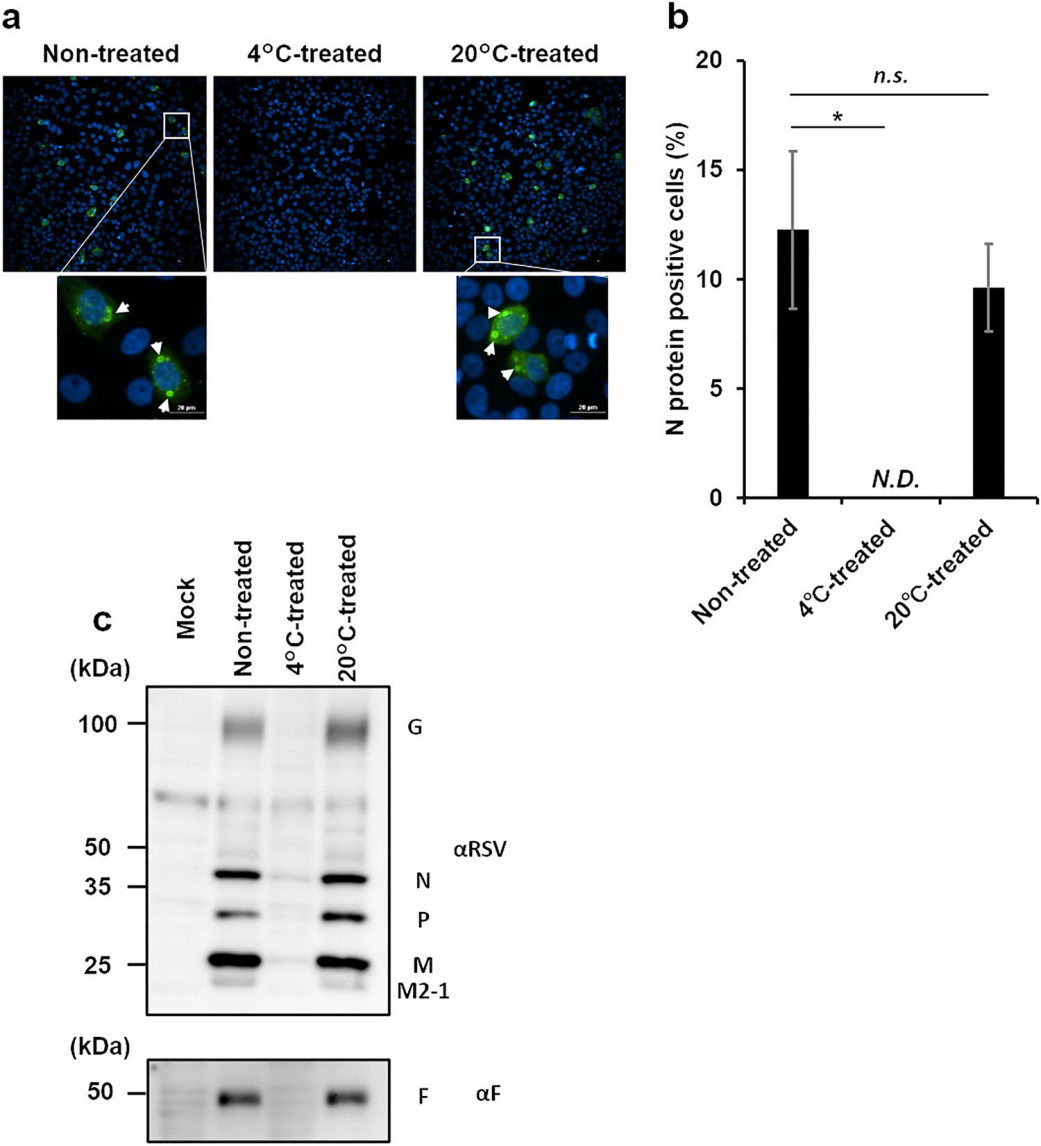
Effect of storage at 4 °C on viral protein synthesis RSV was incubated at 4 °C or 20 °C for 24 h and inoculated onto HEp-2 cells. For the control, RSV was inoculated onto HEp-2 cells without treatment at any temperature. (a, b) At 24 h post-infection, RSV nucleocapsid (N) protein was detected by immunofluorescence staining, and the percentage of N protein-positive cells was calculated. The mean ± SD of ten field views. (c) Intracellular viral protein was detected by western blotting using the anti-RSV F protein and anti-RSV polyclonal antibodies. White allows indicate inclusion bodies. The original blot is presented in Supplementary Fig. 2. *, *p* < 0.05; n.s., not significant.; N.D., not detected.; SD, standard deviation. The significant differences were determined by *t*-tests.

Next, we verified whether the 4 °C-treated RSV taken into the cells could replicate. The viral protein synthesis was investigated by fluorescent immunostaining and western blotting analysis of infected cells with antibodies recognizing viral proteins. Non-treated virus and 20 °C-treated virus formed inclusion bodies (Fig. 2a), the indicator of viral replication, in infected cells 24 h after infection (~ 10% of cells were positive for N protein (Fig. 2b)), and showed the synthesis of viral proteins (N, P, M, M2-1, G, F) in western blotting (Fig. 2c, S2). In contrast, no infected cells and viral proteins were detected by immunostaining and immunoblotting analysis after 4 °C treatment (Fig. 2a–c, S2). These data suggested that 4 °C treatment of RSV affected the viral lifecycle step from uptake into cells to viral protein synthesis.

### Fusion activity of 4 °C-inactivated virus

**Figure 3:**
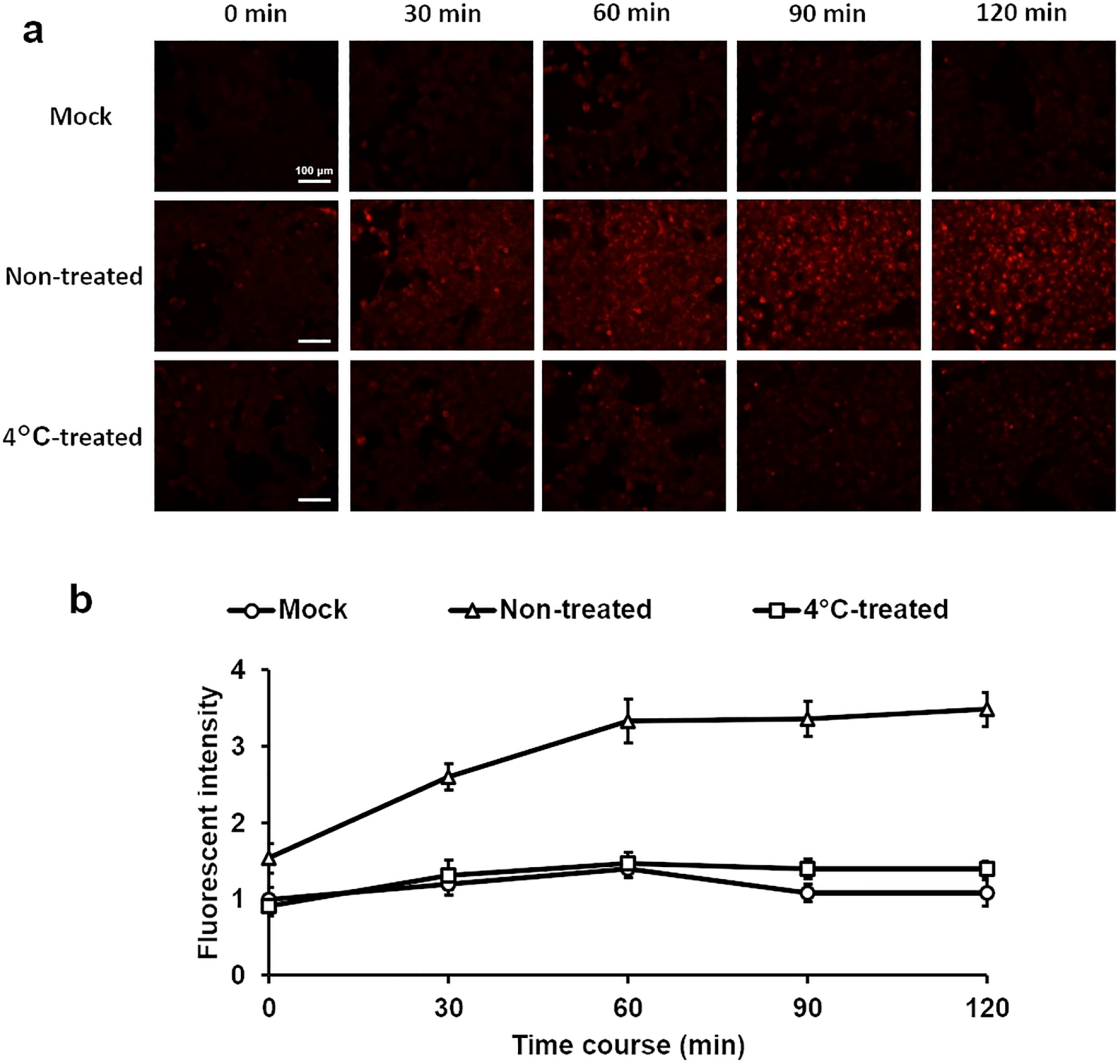
Effect of low temperature storage on the membrane fusion activity of RSV The R18-labeled RSV, with or without 4 °C-storage, was inoculated onto HEp-2 cells, and the membrane fusion activity was measured by a de-quenching assay. For the control, RSV was inoculated onto HEp-2 cells without treatment at 4 °C. (a) Infected cells were incubated at 37 °C and imaged every 30 min. (b) The fluorescence intensity was calculated using ImageJ^47^. The mean ± SD of five fields of view has been shown.

RSV enters host cells via endocytosis and fuses with the intracellular membrane^17–19^. The fusion activity of RSV was evaluated by a previously reported fluorescence de-quenching assay with minor modifications^20,21^. Briefly, 4 °C-treated or non-treated viruses were labeled with rhodamine dye at optimal ratio, followed by purification by spin columns. Purified rhodamine-labeled self-quenching infectious RSV was used. The labeled-RSV was inoculated to HEp-2 cells, and the cells were incubated at 37 °C and observed under a fluorescence microscope at each time point. A high fluorescent signal is detected when the viral envelope fuses with the cell membrane because the self-quenching effect is reduced or eliminated. Although non-specific fluorescence was observed to a minor extent across all samples, bright fluorescence dots indicative of viral and cell membrane fusion were only observed in the control virus samples. (Fig. 3a,b). All these results showed that the inactivation of RSV at 4 °C is due to a loss of the fusion activity.

### Conformational changes in the F protein at low temperatures

**Figure 4:**
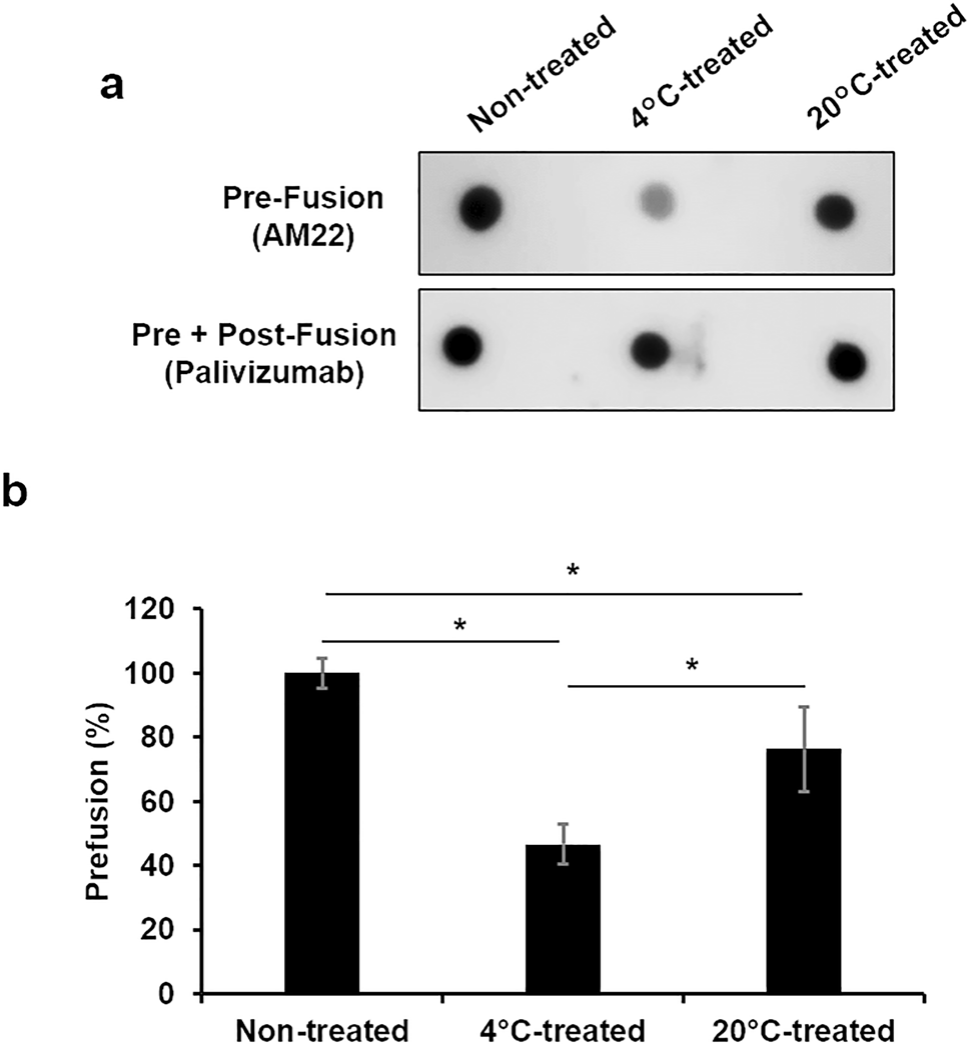
Conformational changes in the F protein at 4 °C or 20 °C The RSV titer was adjusted to 1.0 × 10^5^ TCID_50_/ml, incubated at 4 °C or 20 °C for 24 h, and blotted onto a nitrocellulose membrane. The F protein on the virion was detected using the anti-F monoclonal antibodies, AM22 or palivizumab. For the control, RSV was blotted onto the membrane without treatment at any temperature. (a) Images of detected dots. (b) The intensity of each dot was quantified using ImageJ, and the percentage of the pre-fusion form was calculated according to the formula below^47^. Pre-fusion (%) = (the F protein detected by AM22) / (the F protein detected by AM22 and palivizumab) × 100 Each percentage was normalized to the non-treated virus. The mean ± SD of three dots has been shown. *p < 0.05. The significant differences were determined by ANOVA test.

The membrane fusion of RSV is caused by conformational changes in the F protein from the pre-fusion state to the post-fusion state^22,23^. Similar changes in the F protein can be induced by heating or treating the F protein with formalin in vitro^24,25^. We hypothesized that a similar conformational change occurs in low temperature-sensitive RSV strains at 4 °C. Conformational changes in the RSV/Sendai/851/13 F protein at 4 °C were assessed by a previously reported dot blot method with minor modifications^24^ using structure-specific monoclonal antibodies. Two monoclonal antibodies, palivizumab and AM22, were used. Palivizumab recognizes the epitope, site II, which is common for the pre-fusion and post-fusion forms, and AM22 recognizes the pre-fusion specific epitope, site 0. The detection level of F proteins by palivizumab was not affected by the incubation of virions after 24 h-incubation at 4 °C (Fig. 4a, S3). The detection level by AM22 was not reduced after 12 h-incubation at 4 °C but significantly reduced after 24 h-incubation at 4 °C (Fig. 4a,b, S3, S4). The detection level by AM22 was also reduced at 20 °C. However, the reduction was less severe when compared to 4 °C (Fig. 4a,b, S3, S5). In addition, the N protein was also detected by similar dot blotting (Fig. S3, S5). These results indicate that the ratio of the prefusion protein is required to be at least between 40 and 60% in F proteins for the virus to remain infectious in the storage at 4 °C and that incubation at 4 °C causes more pronounced conformational changes in the RSV/Sendai/851/13 F protein than at 20 °C, impairing the fusion activity.

### Identifying the amino acids involved in low temperature lability of the F protein

**Figure 5:**
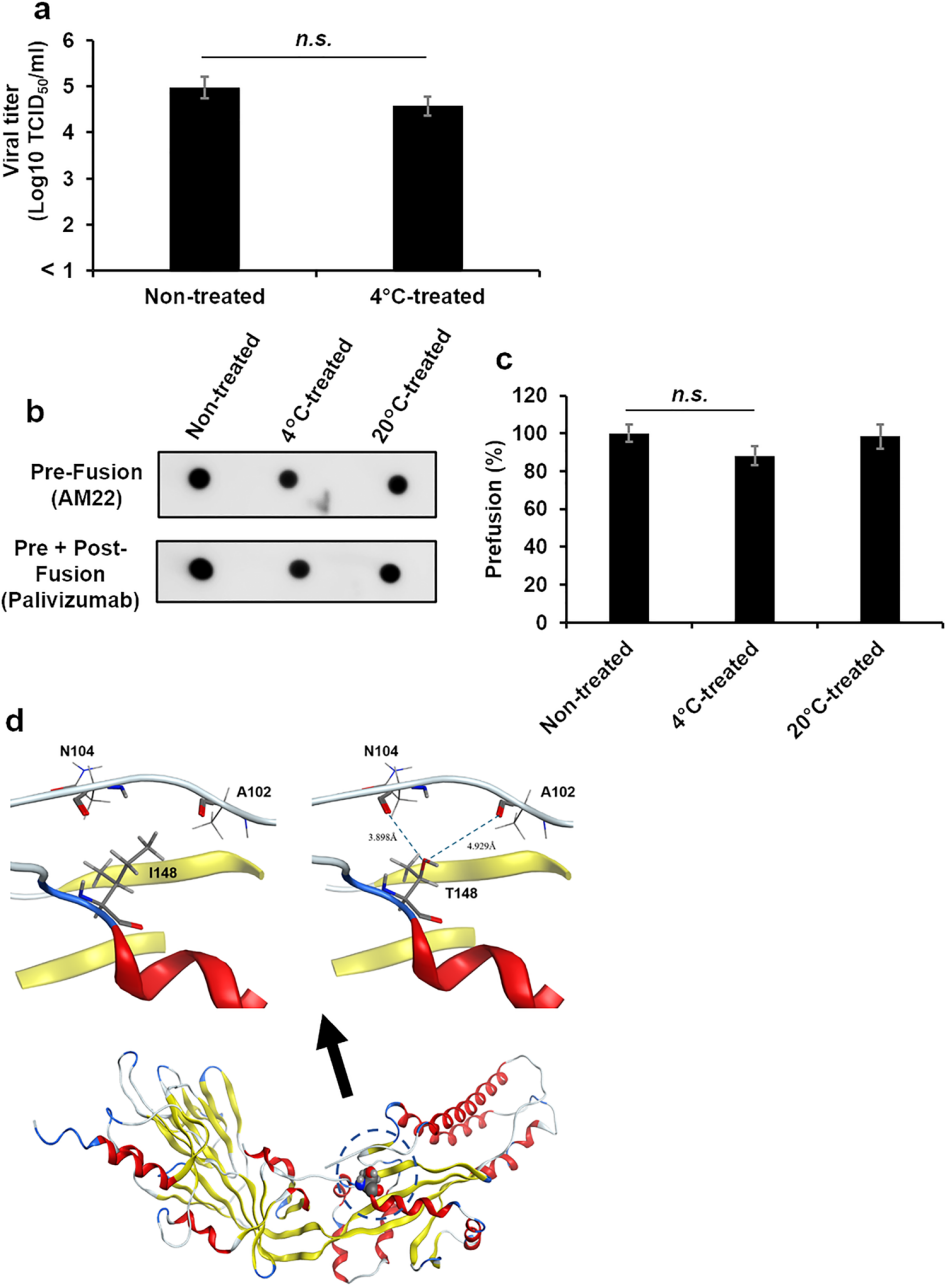
Stability of the infective and pre-fusion form of the RSV/Sendai/851/13 resistant mutant at 4 °C The titer of the RSV/Sendai/851/13 4 °C-resistant variant was adjusted to 1.0 × 10^5^ TCID50/ml, and its infectivity titer and pre-fusion ratio were measured after incubation at 4 °C or 20 °C for 24 h. For the control, RSV was inoculated onto HEp-2 cells or blotted onto the membrane without treatment at any temperature. (a) The viral titers with or without storage at 4 °C are shown. The mean ± SEM of three independent experiments has been shown. (b) The images of detected dots. (c) Each dot was quantified using ImageJ, and the percentage of the pre-fusion form was calculated using the formula below^47^. (d) The structure of the F protein of RSV/B/Sendai/851/13 and its variant were modeled using MOE software package from that of RSV B18537 (PDBID: 6Q0S). Pre-fusion (%) = (the F protein detected by AM22) / (the F protein detected by AM22 and palivizumab) × 100 Each percentage was normalized to control. The mean ± SD of three dots has been shown. n.s., not significant. The significant differences were determined by *t*-tests.

RSV/Sendai/851/13 was incubated at 4 °C for 48 h, followed by inoculation of HEp-2 cells, and the supernatant was passaged three times. The culture supernatant was harvested after the cytopathic effect (CPE) appeared and spread on the cell monolayer. After repeating this procedure three times, a 4 °C-resistant RSV/Sendai/851/13 stock was obtained. Sequence analysis of the parental strain (GenBank accession nos. LC474532.1) and the 4 °C-resistant stock revealed five nucleotide mutations, including a substitution of I148T in the F protein and a substitution of S265L in the G protein (Table 1). The law sequence data showed only a single nucleotide peak at these substitution sites. Therefore, we considered that most of the viruses in the 4 °C-resistant virus stock have these mutations. The obtained 4 °C-resistant RSV/Sendai/851/13 variant retained infectivity after incubation at 4 °C (Fig. 5a). The AM22-recognized structure (pre-fusion-specific epitope 0) was maintained after incubation at 4 °C (Fig. 5b, c). The structure of the F protein of RSV/B/Sendai/851/13 and its variant were modeled using the MOE software package from that of RSV B18537 (PDBID: 6Q0S). There was 97% homology between their amino acid sequences, and ten residues of mutations were introduced (F45L, A103V, C155S, L172Q, F190S, R202Q, L207V, M226K, Q283R, and C290S). The effect of the I148T mutation is shown in Fig. 5d. The difference in chemical structure due to this mutation is the change of the ethyl group to a hydroxyl group: the hydrophobic side chain of I148 cannot form an attractive interaction with the facing residues. However, the hydroxyl group introduced by mutating T148 has hydrogen bonding ability and can form hydrogen bonds with the carbonyl groups of the facing N104 and A102 via the bridging water. Therefore, the formation of stable interactions is thought to lead to low-temperature stability.

**Table 1:** Amino acid substitutions between RSV/851/13 parent and 4 °C-resistant variant.

Nucleotide position (LC474532.1)	Protein	Amino acid substitution
5457 (T → C)	G	S265L
5639 (A → G)	Non-coding	
5644 (C → G)	Non-coding	
6136 (C → T)	F	I148T
14,982 (A → G)	L	Silent

## Discussion

Previously, we reported the inactivation at 4 °C of RSV clinical isolates^13^. Maintaining the infectivity of the virus in clinical specimens is very important for virus isolation, and the results have important implications for laboratory activities in clinical settings. In this study, we aimed to investigate the underlying mechanism of this phenomenon. The results showed that low-temperature treatment, which inactivates RSV, did not affect viral adsorption to or uptake into host cells but reduced membrane fusion ability. These data indicate that the RSV used in this study fused with the cell membrane after endocytosis. Indeed, other studies reported that RSV infects cells via endocytosis pathway^17,19,26^. Further analysis showed that the conformational change induced in the F protein at 4 °C is responsible for the loss of membrane fusion ability and infectivity. Analysis of an RSV/Sendai/851/13 variant suggested that the amino acid residue 148 in the F protein modulates the conformational stability of the F protein at 4 °C and thus for the cold stability of the virus.

Pre-fusion F protein is structurally unstable and can easily undergo a conformational change to the more structurally stable post-fusion form upon stimulation^27^. This conformational change causes membrane fusion between the viral envelope and the host cell membrane. Although the factors involved in the conformational changes of F protein in vivo still need to be studied, conformational changes are induced by heat and formalin treatment in vitro, and conformational changes of F protein are one of the causes of RSV inactivation^24,25^. The data in this study demonstrated that cold temperatures can also induce structural changes in the RSV F protein. After incubation at 4 °C, the proportion of the prefusion form decreased significantly, but approximately 40% remained. The exact requirement for the prefusion form F protein to maintain RSV infectivity remains unclear. However, April et al. also reported that RSV infectivity was significantly reduced when the prefusion rate was below 50% in their dot blotting analysis, indicating that a threshold of approximately 50% determines RSV infectivity^24^. Similar to the conformational change from the pre-fusion to post-fusion form, the F protein conformational change at 4 °C may be irreversible. Indeed, our previous study showed that once the virus is inactivated at low temperatures, its infectivity can not be recovered^13^. Protein inactivation at low temperatures is uncommon, but Heike et al. reported that an HIV protease denatures at low temperatures^28^. The inactivation of the F protein at low temperatures might influence the seasonality of RSV epidemics. While RSV traditionally peaks during the winter, recent trends show peaks during the summer to fall seasons as well, suggesting that the temperature stability of RSV might play a role in these seasonal shifts^29–31^. However, our previous study analyzing the temperature stability of clinical isolates did not find a clear relationship between the low-temperature stability of RSV and the season in which the virus was isolated. These data indicate that further analysis is needed to fully understand the implications of temperature stability on RSV transmission and epidemic trends.

A 4 °C-resistant RSV/Sendai/851/13 variant was obtained from parental strain by treatment at 4 °C and several passages. This variant had an I148T substitution in the F protein and an S265L substitution in the G protein. No substitutions were found in other viral proteins. It is not known whether these amino acid substitutions are due to a viral mutation during the passages or the selection of a variant that was already present in the original stock. At any rate, for a 4 °C-resistant variant virus to occur, three passages in 4 °C were necessary until the ratio of the 4 °C-resistant F protein on a virion surface increased. Many receptor-binding proteins of paramyxoviruses cooperate with F protein to induce membrane fusion^32,33^. The pneumoviral RSV G protein also has cell adsorption ability. Membrane fusion of RSV is mediated only by the F protein and does not necessarily require support of the G protein^34,35^. Therefore, the I148T substitution in the F protein likely plays a vital role in the low-temperature stability of the F protein. However, several studies have reported interactions between the G and F proteins, and it is also conceivable that the S265L mutation in the G protein could contribute to the structural stability of the F protein^36,37^. The F protein of RSV is the major antigen of RSV vaccines, and its structure or fusion state is closely related to the efficacy of the vaccines. Amino acid residue 148 of the F protein is located in the fusion peptide (FP) that inserts into the host cell membrane during conformational change of the F protein^38,39^. Several amino acid sequences of the F protein involved in the thermal stability of RSV have been identified (79, 191, 357, 371, 557)^40^. However, the relationship between the FP and low-temperature stability has not been reported. Our in silico analysis suggested that the substitution of amino acid residue 148 from isoleucine to threonine may increase the structural stability of the prefusion form F protein by forming hydrogen bonds with the amino acid residue 102 or 104 facing 148. Che et al. showed that the amino acid residue 148 is close to amino acid residue 103 and that their interaction may be involved in the conformational stability of the pre-fusion F protein^41^.

A limitation of this study is that only one isolate, RSV/Sendai/851/13, was used to analyze the details of RSV low-temperature stability. Our previous study showed that many clinical isolates are inactivated, to varying degrees, at low temperatures, although some strains were stable at low temperatures. Different strains may have different mechanisms for inactivation by low-temperatures inactivation. However, the present study indicates that the conformational change of F protein at low temperatures is one of the causes of the inactivation of RSV by low temperatures.

In conclusion, this study provides a molecular basis for RSV inactivation at low temperatures. RSV inactivation at 4 °C is due to the loss of membrane fusion activity in the F protein, which cannot maintain its pre-fusion state at 4 °C.

## Materials and methods

### Ethical considerations

All experimental procedures were approved by the Ethics Committee of the Sendai Medical Center, Sendai, Japan and The University of Tokyo.

### Cells

HEp-2 cells were cultured in an incubator (37 °C, 5% CO_2_) in Eagle’s Minimum Essential Medium (Sigma-Aldrich, St. Louis, MO, USA), containing 5% calf serum (Thermo Fisher Scientific, Waltham, MA, USA), 1% fetal bovine serum (FBS) (Thermo Fisher Scientific), 1.7% glucose (TERUMO, Tokyo, Japan), 100 units/ml of penicillin G (Meiji Co., Tokyo, Japan), and 100 µg/ml of streptomycin (Meiji Co.). It is important to note that although HEp-2 cells were originally reported to be derived from human laryngeal carcinoma, subsequent studies have demonstrated that the cells obtained from ATCC are contaminated with HeLa cells^42–44^. They were used to propagate RSV and conduct infection experiments.

### Virus

RSV was isolated from pediatric patients with respiratory symptoms who visited the pediatric clinic of the Sendai Medical Center. RSV/Sendai/851/13 (GenBank accession nos. LC474532.1) used in this study was obtained as a virus stock after cultivating the clinically isolated virus and propagating it for three passages in HEp-2 cells. When CPE had spread sufficiently, the culture medium was collected, centrifuged at 2300 g, and the supernatant was stored as a virus stock. This virus stock was frozen in a dry ice–ethanol bath and stored at − 80 °C.

### Thermal stability assay

Viral titers were diluted from the stock to 1.0 × 10^5^ TCID_50_/ml and the viruses were stored at 4 °C or 20 °C for 24 h. Infectivity was measured in TCID_50_ units using ten-fold serial dilutions on HEp-2 cells cultured in 96-well microplates (Corning inc., Corning, NY, USA). After inoculation of each dilutant, the plate was centrifuged at 500 g for 30 min, incubated at 34 °C for 5 days, and development of CPE was observed to calculate the viral titer according to the Leed and Mench method^45,46^.

### Attachment and entry uptake assays

HEp-2 cells were infected with RSV at a multiplicity of infection (MOI) of 0.1, incubated at 4 °C for 1 h for the virus to adsorb, and washed with Dulbecco's phosphate-buffered saline (DPBS) (Sigma-Aldrich) five times to remove unbound viruses. RNA was extracted from the cells using the RNeasy Mini Kit (Qiagen, Hilden, Germany). For the uptake assay, the cells were incubated with the virus at 4 °C for 1 h, transferred to 37 °C to allow virus uptake, and treated with 0.5% trypsin followed by washing with DPBS five times to detach and wash away the extracellular viruses. Viral RNA was extracted and quantified using real-time PCR to estimate the amount of virus bound to the cell surface or inside the cells.

### Viral RNA quantification by real-time PCR

cDNA was synthesized from the extracted RNA using ReverTra Ace qPCR RT Master Mix (TOYOBO Co., Osaka, Japan). Real-time PCR targeting the RSV L gene was performed using THUNDERBIRD Probe qPCR Mix (TOYOBO Co.)^13^.

### Immunofluorescence staining

HEp-2 cells were seeded on multi-well glass bottom dishes (Matsunami Glass Ind., Osaka, Japan) and cultured for 24 h before virus inoculation. The cells were infected with the virus at an MOI of 1, incubated at 37 °C for 24 h, fixed with 4% paraformaldehyde (FUJIFILM Wako Pure Chemical Co., Osaka, Japan) at room temperature for 30 min, permeated with 0.5% Triton X-100 (Sigma-Aldrich) at room temperature for 10 min, blocked in a 5% skim milk solution (Meiji Co.) at room temperature for 20 min, and stained with the anti-RSV nucleocapsid protein (130/12H) mouse antibody (1:100) (Sigma-Aldrich), followed by the secondary antibody, Alexa Fluor 488-conjugated anti-mouse IgG (1:1000) (Thermo Fisher Scientific). The wells were mounted in VECTASHIELD® PLUS Antifade Mounting Medium with DAPI (Vector Laboratories Inc., Newark, CA, USA) and observed under a fluorescence microscope. The obtained images were analyzed using ImageJ software^47^.

### Western blotting

HEp-2 cells were infected with RSV at an MOI of 0.1, incubated for 72 h, harvested and lysed with EzApply (Atto, Tokyo, Japan), and boiled at 100 °C for 5 min. Proteins were separated on 4–12% NuPAGE Bis–Tris gels (Invitrogen Co., Carlsbad, CA, USA), transferred to a polyvinylidene difluoride membrane (Invitrogen), blocked with 4% skim milk solution at room temperature for 1 h, rinsed with 1% skim milk solution. The membrane was probed with anti-RSV F protein antibody, Palivizumab (1:1000) (AstraZeneka, Cambridge, UK), or anti-RSV (AB1128) goat polyclonal antibody (1:500) (Sigma-Aldrich) followed by HRP-conjugated anti-human IgG (1:5000) (Thermo Fisher Scientific) or anti-goat IgG (1:10,000) (Thermo Fisher Scientific), and detected using ECL Prime Western Blotting Detection Reagent (Cytiva, Tokyo, Japan). Images were captured using ChemiDoc XRS + (Bio-Rad Laboratories Inc., Hercules, CA, USA) and the intensity of dots was calculated using ImageJ^47^.

### Virus-to-cell fusion assay

After adjusting the RSV titer to 1.0 × 10^5^ TCID_50_/ml, it was incubated at 4 °C. It was labeled with 100 µM octadecyl rhodamine B chloride (R18) (FUJIFILM) at room temperature for 1 h. Unbound R18 was removed by Microspin™ G-25 Columns (Cytiva). HEp-2 cells were infected with the R18-labeled RSV, incubated at 4 °C for 30 min, cultured in DPBS containing 5% FBS at 37 °C, and imaged under a fluorescence microscope at 0, 30, 60, 90 and 120 min post-incubation. Non-treated virus used as control virus. Rhodamine diluted in PBS to the same concentration, and then purified by spin column in the same way was used as mock. Fluorescence intensities in the collected images were estimated using ImageJ^47^.

### Preparing the 4 °C-resistant strain

RSV/Sendai/851/13 that was prone to inactivation at 4 °C was diluted to a titer of 1.0 × 10^5^ TCID_50_/ml, stored at 4 °C for 48 h, inoculated onto HEp-2 cells, and incubated at 34 °C for 7 days. Its culture supernatant was inoculated onto a fresh batch of HEp-2 cells as a blind passage and this was repeated until visible CPE could be observed throughout the cell layer. After the CPE spread enough on the cellular sheet, the culture supernatant was collected and incubated at 4 °C and inoculated onto HEp-2 cells again. This procedure was repeated three times to select 4 °C-resistant RSV. Finally, the culture supernatant was harvested as the seed stock of the 4 °C-resistant RSV strain.

### Sequence analysis

Viral RNA was extracted from the virus seed stock using the Viral RNA Mini Kit and amplified using primer sets (Table 2) and the PrimeScript™ One Step RT-PCR Kit Ver.2 (Takara Bio). The amplicons were confirmed by agarose gel electrophoresis, purified using NucleoSpin® Gel and PCR Clean-up (Takara Bio), and sequenced by sanger sequencing (Fasmac Co., Kanagawa, Japan). The sequencing data were analyzed by the MEGAX software version 10.2.2^48^ and compared with parental RSV/851/13 sequence from GenBank (LC474532.1).

**Table 2:** Primer sets for sequencing whole genome of RSV/Sendai/851/13.

	Forward primer (5’-3’)	Reverse primer (5’-3’)
RSV-1	aaatggggtgcaattcactgag	tgcatggtggtgttgtcatttg
RSV-2	tcatcaaagggaaatggggca	gccccaatttatgttaccggc
RSV-3	agcatcaactcaaccccaaaga	ccatcacgagccgaagtagg
RSV-4	tctcaccccaagtgataaccc	ggagccttcgtgaagcttgt
RSV-5	aggacctacttcggctcgt	atttgagccagcacagcact
RSV-6	agcagatctctacgcccaaag	agggccaaaatttgcttgtgaa
RSV-7	gccagacctagagtgcgaata	ggcagtgcgttgattcttgt
RSV-8	atggcatcacaaaaccatgcc	gtgttttcgggggtggttga
RSV-9	cccacaatccactgtgctcg	gttgttgacagctggtgtgtt
RSV-10	tgagtgctttaagaacaggttgg	aggtgtcgttacacctgcat
RSV-11	acagagttgtcgcatttcca	tgccttccagcttgttgacata
RSV-12	tgcatccaacaaaaatcgtgga	tcagcctgtgctgtgatgtt
RSV-13	tgctgaccaccaatcccaaa	agcacgtctgctggtaatcg
RSV-14	gcaaaccatccatctgctcaa	tcacctgaattgttgttgggct
RSV-15	agcatgtcctcgtctgaaca	tccgcatctcaaccctaagc
RSV-16	tgacatggaaagacatcagcct	tcacgatagaaccgcaatcct
RSV-17	tggcccactttaaggaatgcta	gtttttgacaccagccctcaa
RSV-18	actgcatggggtacaatctct	tcggagttgcagagcaattt
RSV-19	aagtcctgagagtagggtccat	aaagcctgtggatccctcat
RSV-20	acattcagtgttcgtgttgagc	atgacagtccaagtgttccagt
RSV-21	ggtaggttcatctacgcagga	tcagttatatacccctctccccaa
RSV-22	tctggatcccacatcaactct	taaagcttctggggttgggtg
RSV-23	ccgtatgcccttgggttgtt	gcatcgcagacaaagatgct
RSV-24	accattcctgctacagatgcaa	gcttcttgagctcattggttgt
RSV-25	gctggacgtaatgaagtattcagc	tgcacatgtgtgattgttagttt

### Dot blotting

RSV/Sendai/851/13 was diluted to a titer of 1.0 × 10^5^ TCID_50_/ml, stored at 4 °C or 20 °C for 24 h, and 2 µl of the suspension was blotted onto a nitrocellulose membrane (Bio-Rad). The membranes were dried, blocked with 4% skim milk solution at room temperature for 1 h, rinsed with 1% skim milk solution, probed either with the anti-pre-fusion antibody AM22 (1:500), kindly provided by Dr. Hougiri (Mitsubishi Tanabe Pharma Co., Osaka, Japan), or the anti-pre-fusion + post-fusion antibody Palivizumab (1:1000), anti-RSV N protein (130/12H) mouse antibody (1:1000). Probed membranes were incubated with HRP-conjugated anti-human IgG (1:5000) (Thermo Fisher Scientific) or HRP-conjugated anti-mouse IgG (1:10,000) (Thermo Fisher Scientific) and detected using ECL Prime Western Blotting Detection Reagent (Cytiva). Images were captured using ChemiDoc XRS + (Bio-Rad) and the intensity of dots was calculated using ImageJ^47^.

### In silico analysis

The structure of the F protein of RSV/B/Sendai/851/13 and a 4 °C-resistant variant were modeled using MOE software package from that of RSV B18537 (PDBID: 6Q0S). Ten amino acid residues (F45L, A103V, C155S, L172Q, F190S, R202Q, L207V, M226K, Q283R, and C290S) in the F protein of RSV B18537 were mutated, hydrogen atoms were complemented using the Protonate 3D function, and positions of hydrogen atoms were optimized by energy minimization using Amber10: EHT force field.

### Statistical analysis

Data were analyzed using the JMP®Pro 11.2.0 software (SAS Institute Japan, Tokyo, Japan). Data are expressed as the mean ± standard deviation or standard error of the mean. Differences between groups were examined for statistical significance using Welch’s *t*-test. A *p* value less than 0.05 was considered significant. In Fig. 4B, statistical significance was examined by ANOVA test.

### Supplementary Information

Supplementary Figures.

## Data Availability

The genome sequences analyzed in the current study are available from the GenBank under the accession number LC474532.1, https://www.ncbi.nlm.nih.gov/nuccore/1707758916
