# Supplementary material for: Detailed analysis of low temperature inactivation of respiratory syncytial virus
Source: Sci Rep. 2024 May 23;14:11823. doi: 10.1038/s41598-024-62658-z (PMC11116427; doi:10.1038/s41598-024-62658-z)
Supplement: Supplementary file 1 — Supplementary Figures. [file 41598_2024_62658_MOESM1_ESM.pdf]

Detailed analysis of low temperature inactivation of respiratory syncytial virus

Yuki Kitai[1, 2*], Oshi Watanabe[2], Suguru Ohmiya[2], Tomoko Kisu[2], Reiko Ota[2], Kazuyoshi Kawakami[3], Hiroshi Katoh[1], Kaori Fukuzawa[4], Makoto Takeda[1], Hidekazu Nishimura[2*]

[1]Department of Microbiology, Graduate School of Medicine and Faculty of Medicine, The University of Tokyo, Tokyo, Japan

[2]Virus Research Center, Clinical Research Division, Sendai Medical Center, Sendai, Miyagi, Japan

[3]Department of Medical Microbiology, Mycology and Immunology, Tohoku University Graduate School of Medicine, Sendai, Miyagi, Japan

[4]Graduate School of Pharmaceutical Sciences, Osaka University, Suita, Osaka, Japan

Running Head: The analysis of RSV inactivation at 4°C

*Address correspondence to

Hidekazu Nishimura, hide-nishimura@mte.biglobe.ne.jp

Virus Research Center, Clinical Research Division, Sendai Medical Center, Miyagi, Japan

Yuki Kitai, y-kitai@m.u-tokyo.ac.jp

Department of Microbiology, Graduate School of Medicine and Faculty of Medicine, The University of Tokyo, Tokyo, Japan

Supplemental figure 1

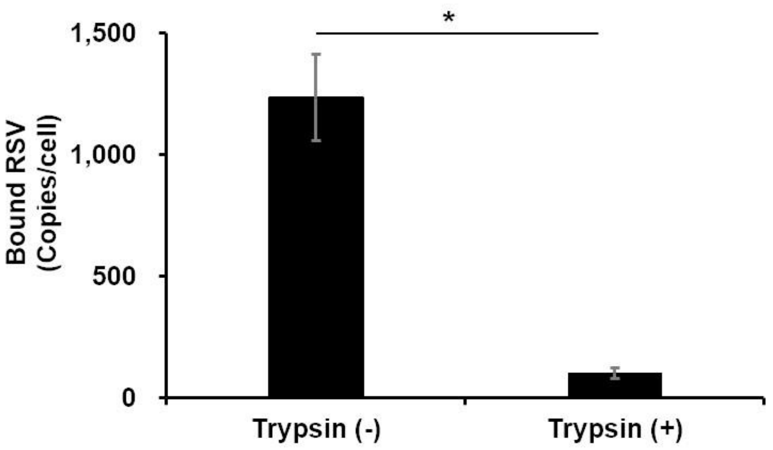

Fig. S1 Trypsin treatment removes virus particles from the cell surface

RSV was inoculated onto HEp-2 cells at an MOI 0.1 and incubated at 4°C for 1 h, followed by washing with DPBS five times. The cells were treated with 0.5% trypsin, followed by washing with DPBS five times to detach and wash away the extracellular viruses. Viral RNA was extracted from HEp-2 cells and quantified using real-time PCR to estimate the amount of virus bound to the cell. The mean ± SD of three wells has been shown. *, $p < 0.05$

Supplemental figure 2

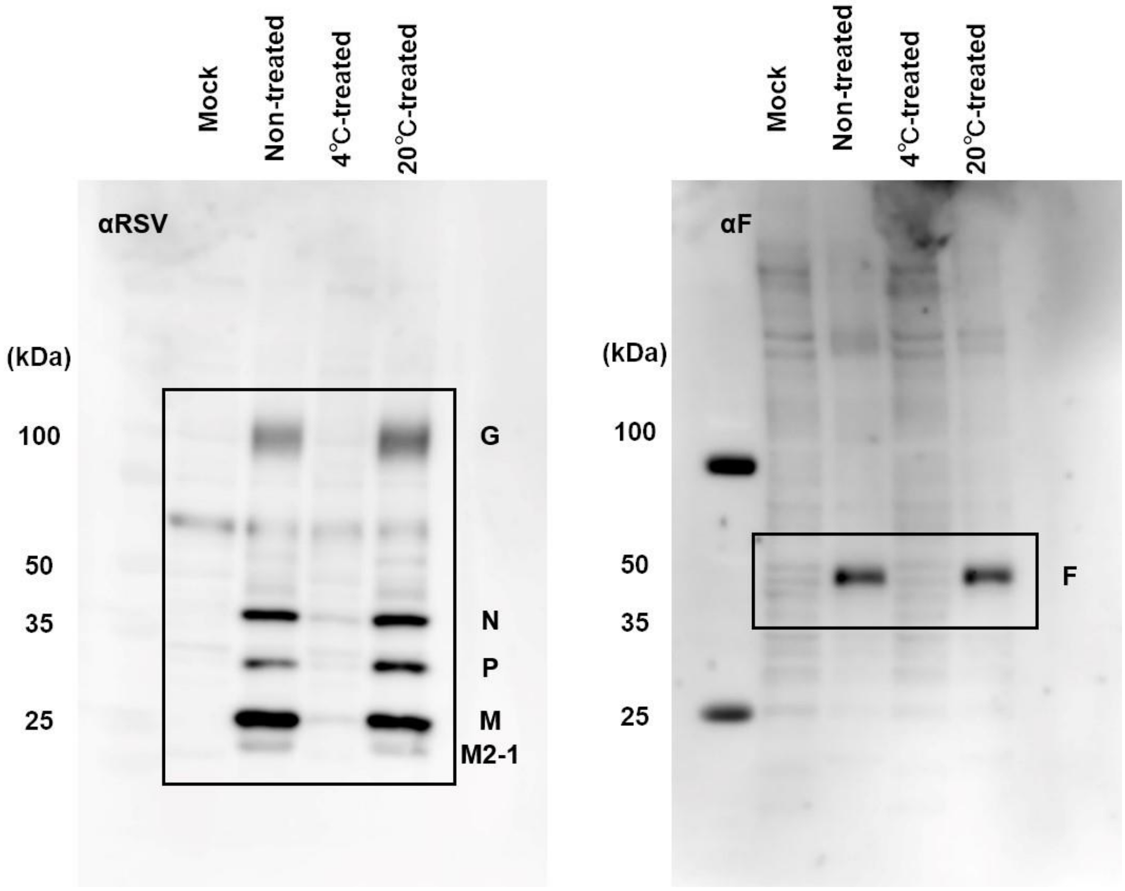

Fig. S2 The original blots of western blotting analysis

The cropped areas are shown in Figure 2. αRSV and αF indicate anti-RSV polyclonal antibodies and anti-RSV F antibodies, respectively.

Supplemental figure 3

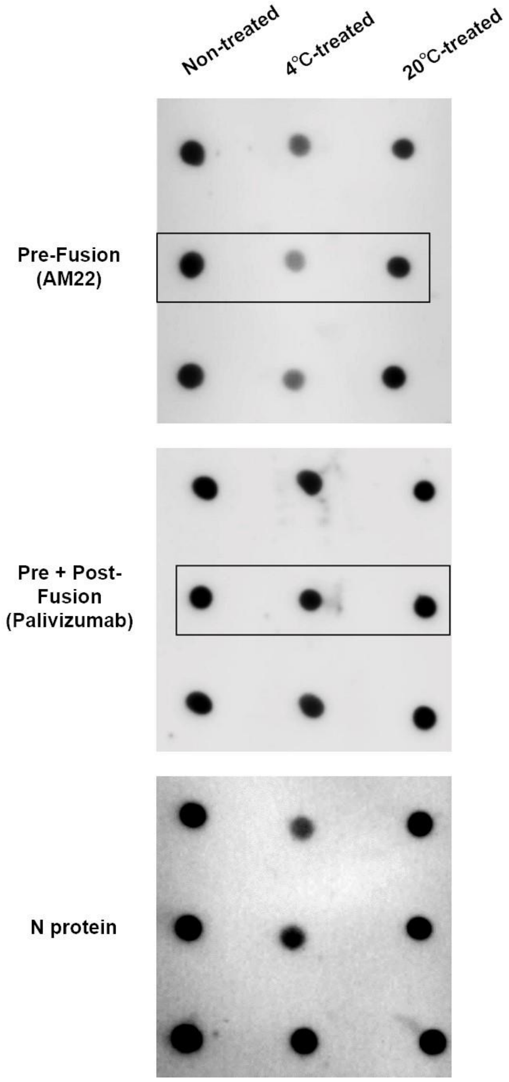

Fig. S3 Original blots of dot blotting analysis

The cropped areas are shown in Figure 4.

Supplemental figure 4

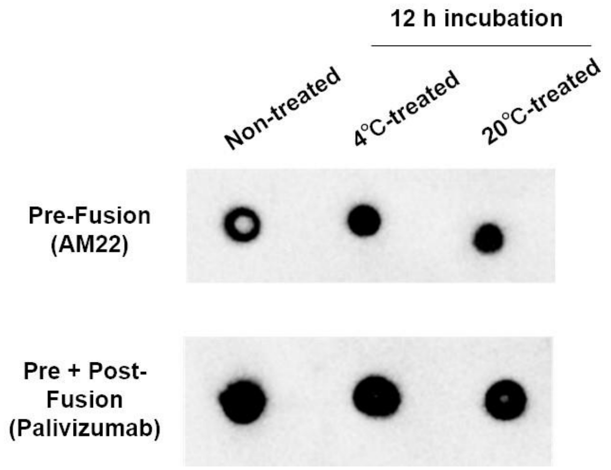

Fig. S4 Original blots of dot blotting analysis

RSV was incubated for 12 hours at 4°C or 20°C, followed by dot blotting analysis.

Supplemental figure 5

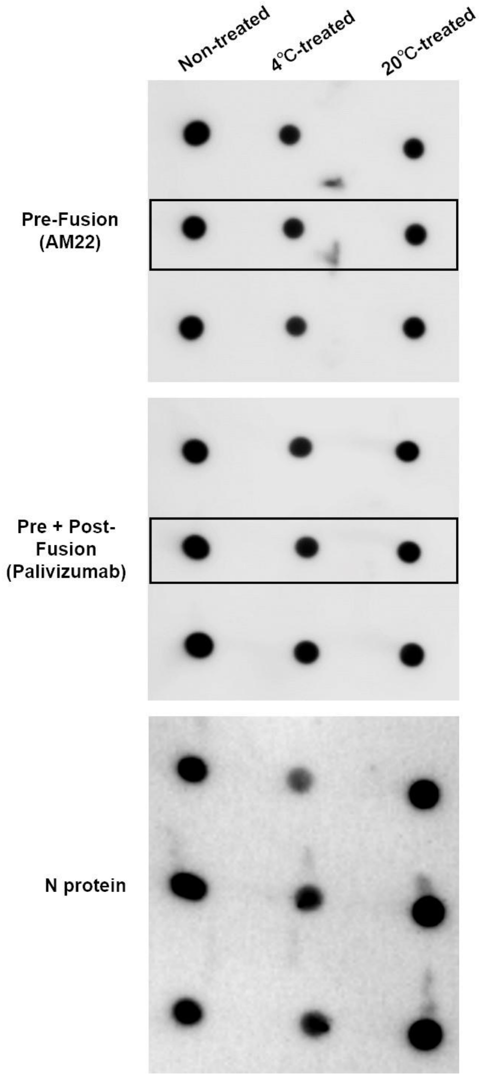

Fig. S5 Original blots of dot blotting analysis

The cropped areas are shown in Figure 5.
